# A Tiny Flexible Differential Tension Sensor

**DOI:** 10.3390/s23041819

**Published:** 2023-02-06

**Authors:** Piotr Z. Wieczorek, Krzysztof Starecki, Krzysztof Gołofit, Maciej Radtke, Marcin Pilarz

**Affiliations:** 1 Faculty of Electronics and Information Technology, Institute of Electronic Systems, Warsaw University of Technology, 00-665 Warsaw, Poland; 2 Talkin’ Things, 02-822 Warsaw, Poland

**Keywords:** tension sensor, ring oscillator, a-IGZO, small signal gain, Miller effect

## Abstract

Modern applications of Internet of Things (IoT) devices require cheap and effective methods of measurement of physical quantities. Cheap IoT devices with sensor functionalities can detect a lack or excess of substances in everyday life or industry processes. One possible use of tension sensors in IoT applications is the automated replenishment process of fast moving consumer goods (FMCG) on shop shelves or home retail automation that allows for quick ordering of FMCG, where the IoT system is a part of smart packaging. For those reasons, a growing demand for cheap and tiny tension sensors has arisen. In this article, we propose a solution of a small flexible tension sensor fabricated in an amorphous InGaZnO (a-IGZO) thin-film process that can be integrated with other devices, e.g., near-field communications (NFC) or a barcode radio frequency identification (RFID) tag. The sensor was designed to magnify the slight internal changes in material properties caused by mechanical stress. These changes affect the dynamic electrical properties of specially designed inverters for a pair of ring oscillators, in which the frequencies become stress-dependent. In the article, we discuss and explain the approach to the optimum design of a ring oscillator that manifests the highest sensitivity to mechanical stress.

## 1. Introduction

Existing methods for measuring strain in integrated circuits (ICs) commonly use silicon piezoresistors as sensing elements, thereby, giving rise to a variety of microelectromechanical system (MEMS) solutions [1]. A different, possibly more sensitive method of measuring strain using regular complementary metal oxide semiconductor (CMOS) technology is based on measuring gate-induced drain leakage as a function of strain (denoted as ϵ); however, this method sacrifices linearity while possibly requiring mechanical stress bias, which might be difficult to achieve under a standard technological process [2].

Despite the low cost and relative popularity of the a-IGZO fabrication process, there is limited literature concerning mechanical strain sensors using this technology, whereas most authors investigating a-IGZO properties have focused on the mechanical stress impact on the durability and electrical characteristics [3,4,5,6]. Furthermore, many other methods utilized for sensor manufacturing commonly make use of non-standard materials, such as pressure-sensitive rubber [7], polydimethylsiloxane (PDMS) film [8] or polyvinylidene fluoride-trifluoroethylene (P(VDF-TrFE)) [9], driving up the manufacturing cost.

A similar issue arises when considering nanomaterial-based sensors [10], as these would require significant changes to the fabrication process to integrate them with standard semiconductor technology. A possible alternative could be to use a metal strain gauge, incorporating it as a part of an RFID inlay and bonding it with the IC—this poses separate issues related to miniaturization [11]. The aspect of converting the sensor’s output to a quantity usable in a digital circuit is also usually omitted.

While the strain-dependent resistance or capacitance is easy to measure in a laboratory setting, it requires additional mixed-signal circuitry to incorporate into a larger digital system. An interesting solution to this problem can be found in [12], where the variable mobility of charge carriers affects propagation delay within ring oscillators. This provides a frequency output, which can then be measured using a simple digital counter.

There are several articles in which the authors reported mechanical stress-induced changes of a-IGZO material, though these are presented in terms of changes in the electrical performance of flexible displays, rather than sensors applications. The authors in [13] reported the electrical performance and stability of a-IGZO TFTs on a polyimide substrate for various degrees of mechanical stress. In the article, the authors created variable mechanical stress by changing the bending radius of a device under test. The experiments showed that the mechanical strain increased the sub-gap Density of States (DoS), which, in turn, caused the parameter deterioration of a-IGZO devices [13,14]. Geng et al. in [9] built an a-IGZO piezoelectric sensor; however, their solution required integration with a P(VDF-TrFE)/PZT composite piezo-capacitor. In that solution, the pressure caused by external force changed the top-gate potential, leading to a change in the transistor threshold voltage.

In this article, we propose a practical use of a-IGZO stress-induced parameter deterioration in a stress (tension) sensor. For this purpose, we use a process described in [15] to manufacture a sensor consisting of a pair of specially designed ring oscillators, with each of the oscillators exposed to a different mechanical strain due to the physical construction of the sensor. The chosen process, uses patterned layers of N-type metal-oxide thin-film transistors (TFT) and resistors deposited on a polyimide substrate. The transistors in such a technology manifest a very low leakage current; therefore, they are suitable for low-power applications, such as RFID or memory designs [16].

The technology offers 200mm flexible polyimide wafers with four routable metal layers. The other advantages of the process are as follows: the use of conventional processing equipment (adapted to produce flexible polyimide substrate electronics), low mask and wafer production costs in high-volume production. Moreover, the process offers a minimum channel length of 0.8μm and 30μm thickness of the whole wafer, whereas the minimum supply voltage is only 3V [15].

The advantages of the flexible IGZO process result in faster design and higher customization capabilities, allowing fitting ICs to exact products and needs. Lower chip thickness and elasticity are also beneficial in the later stages of integration. They give the chip more durability while incorporating it into the inlay and the final product, raising the final production yields.

The rest of the article is organized as follows: Section 2 covers all the issues related to the characterization of mechanical stress influence on electrical parameters, the design of the sensor, and its optimization. In Section 2.1, we describe various test structures used to estimate the stress-induced variability of electrical parameters. Based on these findings, we approximate to what extent the strain affects particular operating regions of N-type metal oxide amorphous semiconductor field effect transistor devices available in the process and propose the strain-sensitive ring oscillator structures in Section 2.2. Section 2.3 covers the electrical macromodel optimization of the proposed sensor, whereas Section 3 shows the circuit frequency measurement results for various strains. The same section explains the difference between the expected and measured circuit sensitivity to strain.

## 2. Materials and Methods

The implementation of the flexible tension sensor that can be designed in the same process as the microcontroller or an RFID tag requires a suitable flexible substrate TFT-based technology [15,17,18]. Despite the obvious advantages of flexible technologies, such as their low overall production cost [5,19], rapid tapeouts, and flexibility, the flexible substrate TFT-based processes have some major drawbacks that limit their functionality, i.e., a limited number of metallization and interconnection layers, limited carrier mobility, and relatively wide parameter tolerances when compared to standard CMOS monocrystalline processes.

The flexible TFT a-IGZO 0.8μm process that was chosen for the target implementation provides only three fundamental circuit elements, i.e., an N-type metal oxide amorphous semiconductor field effect transistor (NMOS), a resistor, and a metal oxide semiconductor capacitor. Therefore, the design of the discussed sensor was demanding and highly process-dependent. However, the basic underlying assumption was that the sensor should be as simple as possible. It should be implemented with the use of standard cells (logic gates used for other designs in this technology), immune to local process variation, and the output tension-dependent quantity should be easily processed by a logical sequential device (e.g., a microprocessor). For the above reasons, a ring oscillator with a stress-dependent frequency output was chosen as the target design.

### 2.1. Influence of Mechanical Stress on Electrical Parameters

**Figure 1:**
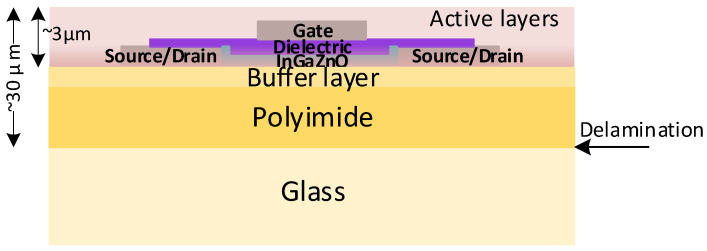
Schematic diagram of the IGZO structure stack [13,19,20].

The example cross-section of active layers and a polyimide substrate in the process chosen for a target design is shown in Figure 1.

The wafers are manufactured on a glass substrate and delaminated after production. After the delamination, the thickness of the active layers forming the electronic devices is still very small (less than 3μm) in comparison to the thickness of the whole flexible wafer (30μm) [15]. In order to investigate the electrical changes, induced by the mechanical stress to the basic parts, such as NMOS transistors, thin-film metal-oxide resistors, and MOS capacitors, we designed and implemented test series for each component type. These results were used to investigate and approximate the stress-induced changes in basic component model parameters.

**Figure 2:**
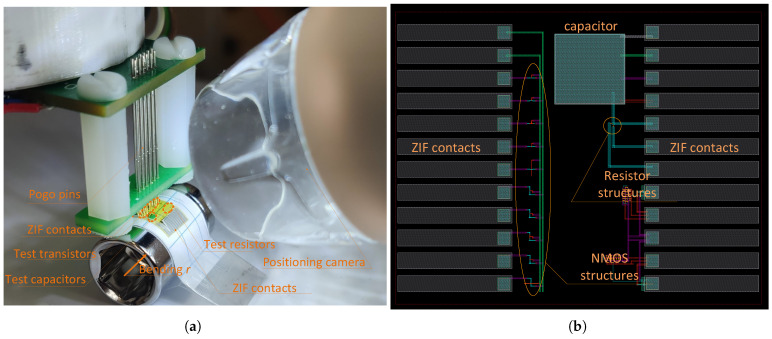
Test structure attached to a bending jig (**a**) and layout of the test structures (top view) (**b**).

The test structures with basic components were subjected to the mechanical stress (compression) caused by bending on a jig of a particular radius, whereas the electrical connections to the bent wafers were made with the use of either a 12 contact 500μm pitch zero insertion force (ZIF) connector or pogo pin contacts as shown in Figure 2a. The layout of the test structure that allowed us to evaluate stress-caused changes in the transfer characteristics of an NMOS, in resistance, and in-MOS capacitance is shown in Figure 2b. It was extremely important to verify the impact of the width- (*W*) to-length (*L*) ratio (W/L) on the transfer characteristics of mechanically stressed devices.

The impact of mechanical stress on the transfer characteristics of NMOS transistors with various W/L ratios can be further used to maximize the whole sensor circuit sensitivity. Therefore, transistors with the following W/L ratios were implemented: 3/0.8 (smallest permitted dimensions), 3/8, 3/80, 30/0.8, and 300/0.8 (corresponding sizes given in μm) and their transfer characteristics, i.e., $I_{D}=f\left(U_{GS}\right)$ were measured both on a flat surface and on the bending jig (cylinder of a certain radius) leading to a compressive strain ϵ=0.2%. Resistance and capacitance measurements were also performed with W=4μm, L=40μm resistive tracks and W=L=1500μm capacitor structures, respectively.

In order to measure NMOS transfer (*I*–*U*) characteristics, we used an Agilent 33600A (Agilent Technologies, Santa Clara, CA, USA ) function generator acting as a programmable gate-source voltage source ($U_{GS}$), whilst the Keysight DSOS604A oscilloscope (Keysight, Santa Rosa, CA, USA) registered the voltage drop on a reference drain resistors, corresponding to the drain current $I_{D}$ of a flat device and $I_{D_{\mathrm{stress}}}$ current of the same transistor subjected to a compressive strain ϵ=0.2%.

During the measurements of transfer characteristics (both $I_{D}\left(U_{GS}\right)$ and $I_{D_{\mathrm{stress}}}\left(U_{GS}\right)$), the $U_{GS}$ voltage varied in the 0–2.2V range, whereas the indirectly measured current ranged from $I_{D}=10\;\mu A$ for W/L=3/80 to $I_{D}=10\;\mathrm{mA}$ for W/L=300/0.8 at the operating point of $U_{DS}=5\;V$, and maximum $U_{GS}=2.2\;V$. Therefore, during all of the measurements, the transistors operated in the saturation region, and the measurements of the transfer characteristics are approximated by Equation (1):

(1) $$I_{D}=\frac{K_{n}}{2}{\left(U_{GS}-U_{T}\right)}^{\alpha }\;$$

where $K_{n}=\frac{\mu C_{ox}W}{L}$, $\mu \approx 7\;\frac{cm^{2}}{\mathrm{Vs}}$ is the estimated average carrier (electron) mobility; $C_{ox}\approx 2.6\;\frac{\mathrm{fF}}{\mu m^{2}}$ is the estimated dielectric capacitance (see Figure 1) per unit area of unstressed NMOS transistors (pristine conditions after detaching form the carrier glass); $U_{T}$ corresponds to a threshold voltage, at which a significant increase of drain ($I_{D}$ or $I_{D_{\mathrm{stress}}}$) current is observed; and α ranges from 2 to 2.3 [13,21]. However, in the case of our experiments, the square law, i.e., α=2 was functional.

**Figure 3:**
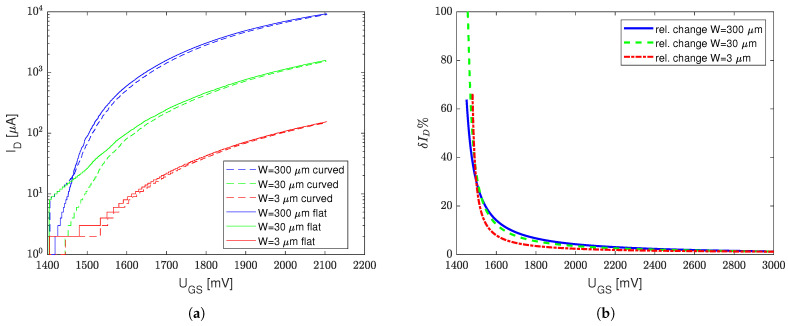
Transistor transfer characteristics for unstressed and stressed conditions (compressive strain ϵ=0.2%). Transistors with the minimum channel length (**a**) and a relative difference of $I_{D}$ and $I_{D_{\mathrm{stress}}}$ transfer characteristics (with and without compressive strain) at various $U_{GS}$ (**b**).

**Figure 4:**
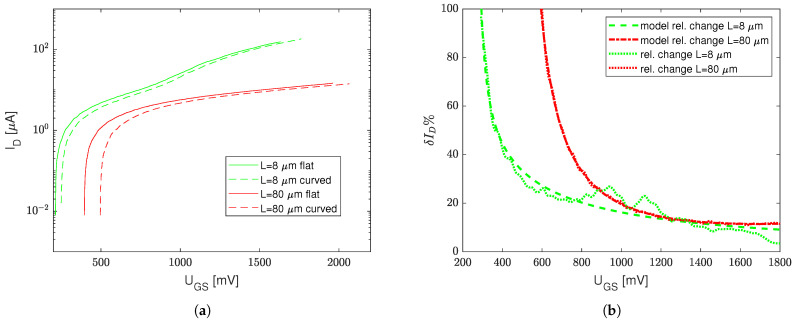
Transfer characteristics of stressed (compressive strain ϵ=0.2%) and unstressed transistors with minimum channel width (**a**) and their relative difference (**b**) at various $U_{GS}$.

The measurement results approximated with Equation (1) are presented for both mechanically stressed (bent on a 3mm diameter cylinder resulting in compressive strain ϵ=0.2%) and unstressed (flat) transistors with the minimum possible channel length, i.e., 3/0.8, 30/0.8, 300/0.8W/L (see Figure 3a), and the minimum possible channel width, i.e., 3/8, 3/80W/L (see Figure 4a).

In order to better understand the stress impact on transfer curves, a relative drain current difference $\delta I_{D}{|}_{\epsilon =0.2\%}=\frac{I_{D}\left(U_{GS}\right)-I_{D_{\mathrm{stress}}}\left(U_{GS}\right)}{I_{D}\left(U_{GS}\right)}$ for $U_{GS}\in \left\{0,\;2.2\right\}\;V$ is shown in Figure 3b and Figure 4b. Two extreme cases were taken into the consideration, i.e., the strain impact for the minimum available transistor length L=0.8μm (Figure 3b) and the minimum transistor channel width W=3μm (Figure 4b).

Due to the local process variation (that randomly affects $U_{T}$) and different ranges of magnitudes of $I_{D}$ (and $I_{D_{\mathrm{stress}}}$) for high and low W/L ratios, the measurement results of the transfer characteristics are separated between the devices with minimum *L* (Figure 3a) and minimum *W* (Figure 4a). However, one can see that mechanical stress affects the transfer characteristics primarily for relatively low $U_{GS}$ voltages.

This results from the different influence of two parameters in Equation (1), i.e., the threshold voltage $U_{T}$ and $K_{n}$ (which is mobility μ dependent) on $I_{D}$ (and $I_{D_{\mathrm{stress}}}$). The strongest influence on the mechanical stress (compression) is visible when the $U_{GS}$ voltage is close to $U_{T}$; hence, slight variations of $U_{T}$ (resulting from the mechanical stress) can cause huge fluctuations of $I_{D}$. However, when $U_{GS}-U_{T}\gg 0$, the mechanical-stress-induced $U_{T}$ increase is masked at high transconductances, i.e., when $g_{m}=\frac{dI_{D}}{dU_{GS}}{|}_{U_{GS}\gg U_{T}}$.

This phenomenon results from a relatively low linear influence of the $K_{n}\left(\mu \right)$ drop on the $I_{D}$ current, opposite to the $U_{T}$ increase, which, in turn, heavily affects the second order term in Equation (1) when $U_{GS}\approx U_{T}$. Table 1 presents $K_{n}$ and $U_{T}$ values obtained with various transistor sizes for both the stressed (bent on a 3 mm cylinder) and unstressed conditions.

The impact of the stress-induced $U_{T}$ increase on $I_{D}$ ($I_{D_{\mathrm{stress}}}$) is highest for relatively low quiescent points. Regarding the operation of a ring oscillator, acting as a tension sensor, in which the frequency should strongly depend on mechanical stress, the above considerations enforce the operation of transistors slightly above $U_{T}$. Moreover, the switching time of a transistor in an oscillator should be at its maximum near the low $U_{T}$ operating region.

According to Table 1, one can see that, in the case of either low *L* or low *W* transistors, $K_{n}$ is not a linear function of the W/L ratio—this phenomenon is not further analyzed in this paper. Nevertheless, from the sensor design point of view, the most important thing is that the highest stress impact on $I_{D}$ was observed for high L/W devices. In the case of a stressed transistor of W/L=300/0.8, the relative current drop for $U_{GS}=2\;V$ was 4.5%, whereas, in the case of the W/L=3/80 transistor, this relative current drop reached up to 10%, which was close to the W/L=3/8 transistor with a 9% drop.

We also investigated the behavior of resistors and capacitors subjected to compressive strain, as investigated previously with transistors. In the case of resistors (see Figure 2b), we observed a relative resistance increase caused by the piezoresistive effect of 4.1% for a 0.2%, 2.5% for a 0.12% strain and 0.8% for a 0.07% strain (ϵ). The resistance vs. stress dependence appears to be quite linear; therefore, we exclude any defective effects, such as contact resistance degradation at higher strains.

In the case of the capacitors with a thin dielectric used as the plate separator, we expected low capacitance stress-induced changes resulting only from the material deformation. While bending the capacitor changes its geometric shape, neither the metallization area nor the dielectric width (i.e., the parameters determining the capacitance) change significantly, which leads to the conclusion that the stress-induced capacitance changes cannot be large. For the applied mechanical stresses, the observed effect was predictably small, similar to the change in the dimensions of the structure resulting from the ϵ, i.e., the maximum relative decrease of capacitance was 0.24%, for ϵ≈0.2%.

According to the above considerations, one can see that the mechanical compression of the flexible wafer caused the NMOS drain current to drop and the resistance to rise, whereas the capacitors maintained almost constant parameters. Based on these results, we made adjustments to the Cadence SPECTRE environment models of the NMOS, resistor, and capacitor components. In this way, we obtained an appropriate model version with adjusted parameters for the mechanically-stressed devices. Therefore, we were able to optimize the electrical behavior of more sophisticated circuits in two extreme conditions—stressed and unstressed.

### 2.2. Design of a Ring-Oscillator-Based Sensor

Mechanical compressive stress shifts the $U_{T}$ voltage towards higher values and slightly decreases $K_{n}$ due to a slight decrease in the mobility μ. Similar results were reported in [22], where the authors explained the $U_{T}$ shift by changes in the Fermi function. In contrast, a change in the electron-lattice interaction explained the mobility changes due to variations in the interatomic distance [22]. Moreover, the authors in [5,6] reported that, under tensile strain, an opposite shift of $U_{T}$ and the increase in mobility can be observed, which are caused by the rise of the midgap DoS and donor-like and acceptor-like states [23].

**Figure 5:**
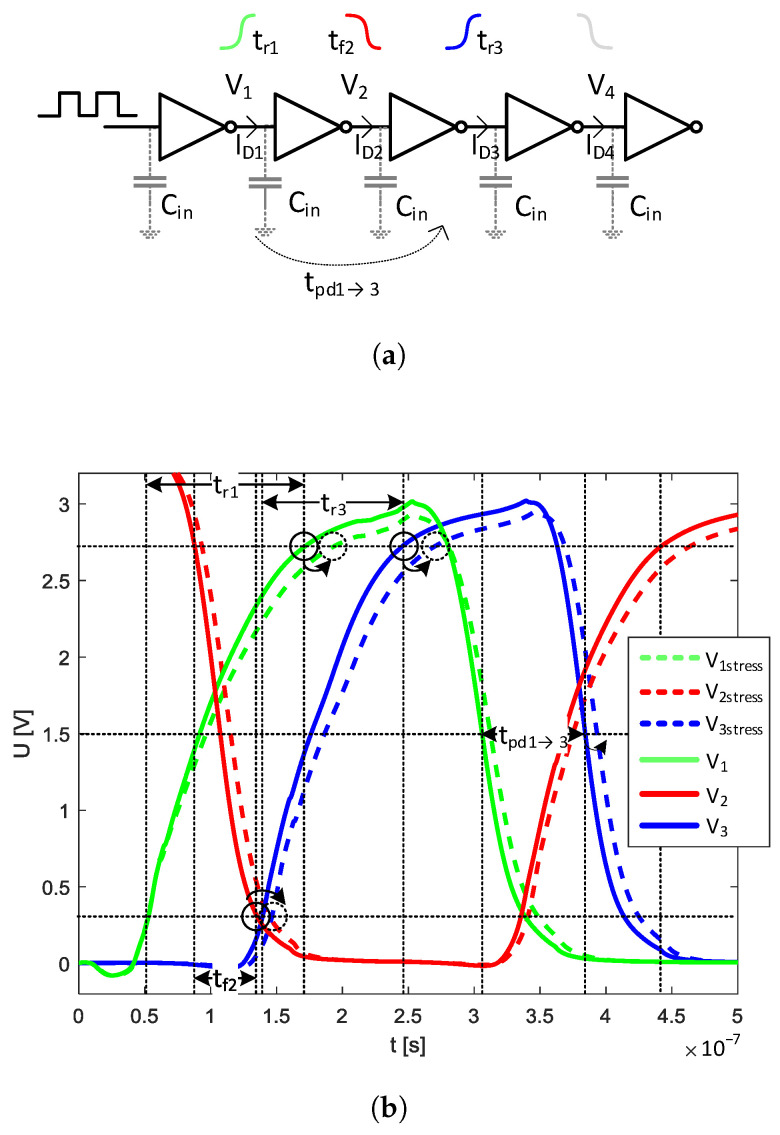
Explanation of the mechanical stress influence on $t_{pd}$ and the frequency performance of logical gates: (**a**) example circuit consisting of a five-stage cascade of inverters and (**b**) output waveforms at the corresponding inverter stages (appropriate 10%, 50%, and 90% levels are marked on the waveforms, and the example propagation delay $t_{pd1\to 3}$ between a pair of inverters).

The $U_{T}$ voltage, drain current, and drain resistance define the static voltage characteristics ($U_{\mathrm{out}}\left(U_{\mathrm{in}}\right)$) and output current characteristics ($I_{D}\left(U_{\mathrm{in}}\right)$) of logic gates implemented in either pseudo-CMOS or MOS resistor–transistor logic (RTL) [24,25]. Considering the capacitive character of the logical gate input, a change of the drain current $I_{D}$ or the drain resistance influences the gate’s output slew rate, i.e., $\frac{dV(t)}{dt}=\frac{I_{D}\left(t\right)}{C_{\mathrm{in}}}$ in the case of a cascade connection (see Figure 5a).

This means that $\delta I_{D}$ caused by mechanical stress affects the dynamic performance of a logic circuit, changing its output rise and fall times ($t_{r}$ and $t_{f}$, respectively, as denoted in Figure 5b). These times, in turn, influence the overall propagation delay ($t_{pd}$) of logical gates (or inverters), thereby, affecting the maximum frequency of operation (see Figure 5b). Moreover, the observed stress-induced $U_{T}$ shift delays the gate turn-on time, leading to even higher $t_{pd}$.

This phenomena can be used for mechanical stress measurements, where the stress-induced $t_{r}$ and $t_{f}$ changes affect the $t_{pd}$ of inverters connected in a ring, leading to a stress-variable frequency of oscillation. Figure 5b also shows an example of the propagation delay ($t_{pd1\to 3}$) increase between the first and third stage of a cascade formed of inverters with a stress-dependent $I_{D}$ and $U_{T}$.

**Figure 6:**
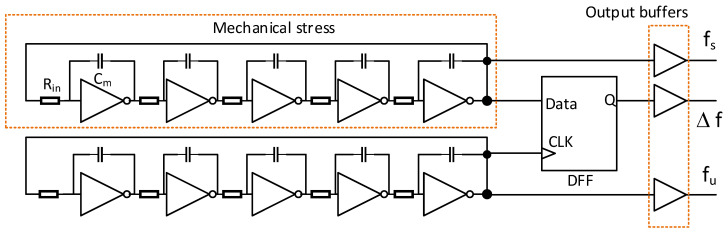
Block diagram of the sensor circuit.

Based on the previous considerations and measurements of electronic circuit elements, we propose a sensor circuit utilizing a pair of identical ring oscillators consisting of logical gates (i.e., inverters) and a frequency mixer built of a D flip-flop (DFF). The block diagram of the device is shown in Figure 6. In the proposed circuit, only one of the ring oscillators is subjected to significant mechanical stress (its frequency is denoted as $f_{s}$), whereas the other oscillator (operating at the $f_{u}$ frequency) is located on a structure in such a way that the material deformation in its neighborhood can be neglected.

In this way, the difference (deviation) of generator (ring oscillators) frequencies $\Delta f=f_{s}-f_{u}$ resulting from stress-induced electrical parameter changes can be observed. In order to obtain a differential frequency at the sensor output, a simple master-slave DFF was implemented, in which a non-zero differential frequency Δf≠0 of the ring oscillators causes a time shift between the active (rising) slopes at the data and CLK inputs. This, in turn, leads to periodic changes of the signal at the DFF output. In this way, the DFF acts as a frequency mixer.

**Figure 7:**
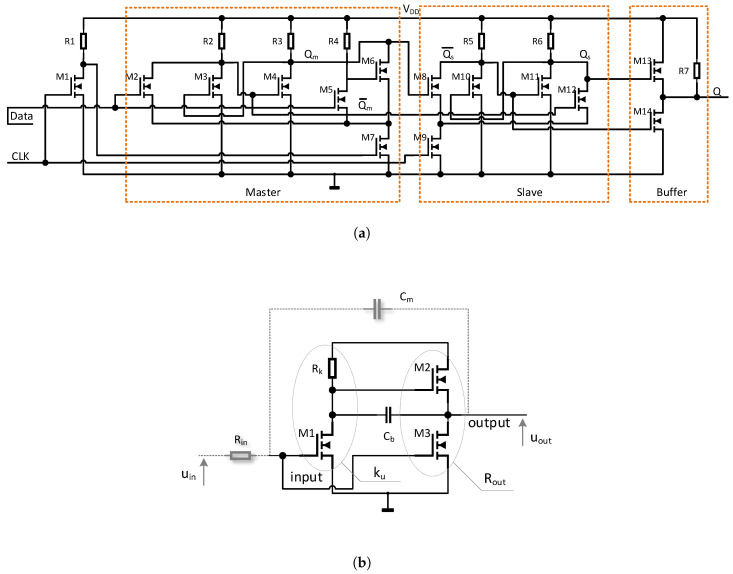
Internal schematics of sensor subcircuits: (**a**) schematic diagram of the D flip-flop used as a frequency mixer and (**b**) schematic diagram of the inverters used in the ring oscillator.

The inverters and DFF were implemented in the pseudo-CMOS logic and RTL, which resulted from the lack of PMOS devices in the a-IGZO process [26]. Therefore, the DFF presented in Figure 7a consists of two jamb-latch stages (master and slave) [27] built with low-power RTL inverters. The data transmission between the master and slave stages of the DFF is differential, and both the $Q_{m}$ and $\bar{Q_{m}}$ output lines are used to reduce the sustain and hold times of the DFF and to keep the energy consumption as low as possible. The DFF output is buffered with a push–pull transistor pair, i.e., $M_{13}$ and $M_{14}$.

Ring oscillator inverters (whose schematic diagram is shown in Figure 7b) were designed to allow adjustment of their small-signal gain $k_{u}$ (the slope of the transfer characteristics) and their output resistance $R_{\mathrm{out}}$ independently. An additional $R_{\mathrm{in}}$ resistance connected in series with the inverters increases the input charge/discharge time constant, which is ϵ-dependent. The proper choice of $k_{u}$, $R_{\mathrm{in}}$ and $R_{\mathrm{out}}$ allowed us to maximize the frequency sensitivity (which is the inverse function of $t_{pd}$) of the ring-oscillator sensor to the mechanical stress.

The inverters are internally coupled using $C_{b}$, in order to obtain the bootstrap effect maximizing the output voltage swing [24]. The external capacitor $C_{M}$ provides the additional Miller effect. Due to the negative $k_{u}$ value, the Miller effect occurs, and the $C_{M}$ capacitance is multiplied at the inverter input. The use of $C_{M}$, together with $R_{\mathrm{in}}$, increases the rise $t_{r}$ and fall $t_{f}$ times as well as the overall propagation delay $t_{pd}$, which, in turn, determines the frequency of the ring oscillators, i.e., $f=\frac{1}{2\cdot n\cdot t_{pd}}$, where *n* corresponds to the odd number of inverters in Figure 6.

The Miller effect is mostly visible when the small signal gain $k_{u}$ of the transfer characteristics reaches its maximum, i.e., when all the transistors in Figure 7b operate in the pentode region. Therefore, the Miller effect significantly reduces $f_{s}$ and $f_{u}$ and, hence, the internal power dissipated by the inverters resulting from charge flow in the internal gate-source $C_{GS}$, gate-drain $C_{GD}$, and parasitic capacitances. In this particular design, the number of inverters was n=5, which corresponded to $f_{u}<100\;\mathrm{kHz}$.

### 2.3. Circuit Analysis and Optimization

In the proposed design, the Miller effect was used not only to reduce the frequency and energy requirements but also to increase the sensor sensitivity to stress-induced parameter changes. The increase of ϵ affects $k_{u}$, due to its sensitivity to $R_{k}$ (see Figure 7b) and, on the other hand, decreases the transconductance $g_{M1m}=\frac{dI_{DM1}}{dU_{GSM1}}$ of $M_{1}$. These two factors mainly affect the internal small signal gain of the first stage of inverter (formed of $M_{1}$ and $R_{k}$), and therefore the gain of the whole inverter becomes strain dependent.

One can see that $M_{2}$ acts as a voltage follower (whose gain is <1) with a dynamic load formed of $M_{3}$; therefore, their impact on $k_{u}$ is negligible in the pentode region, giving $k_{u}\left(\epsilon \right)\approx g_{M1m}\left(\epsilon \right)R_{k}\left(\epsilon \right)$. The relative $I_{DM1}$ stress-induced changes varied from 2% to 100%, whereas $R_{k}$ can vary up to 4.1% for a constant ϵ (see previous subsection). Therefore, a small signal sensitivity of $k_{u}$, i.e., $\frac{dk_{u}\left(\epsilon \right)}{d\epsilon }$ depends mainly on the parameters of $M_{1}$; hence, properly adjusted $L_{M_{1}}$ and $W_{M_{1}}$ with a transistor in a proper region of operation causes the stress-induced $I_{DM1}$ changes to dominate over the $R_{k}$ changes. Summarizing, the $M_{1}$ dimensions *L* and *W*, are crucial for the circuit sensitivity to mechanical stress.

The variable gain $k_{u}\left(\epsilon \right)$ also magnifies $C_{M}$ at the inverter input depending on the strain. The Miller multiplied $C_{M}\left(1+\right|k_{u}\left(\epsilon \right)\left|\right)$ capacitance, together with $R_{\mathrm{in}}\left(\epsilon \right)$, influences the $t_{pd}$ of the inverter. Moreover, the internal output resistance $R_{\mathrm{out}}$ of the inverter, formed by the parallel connection of drain-source (channel) resistances of $M_{2}$ and $M_{3}$, varies with the strain. The higher the compressive strain, the higher the channel resistances (lower transconductances) of $M_{2}$ and $M_{3}$ [13], leading to an additional increase in $t_{r}$ and $t_{f}$ as well as the resultant propagation delay ($t_{pd}$) of the inverter.

One can see that the total strain impact on the $t_{pd}$ of the inverter is extremely difficult for symbolic analysis. In order to perform an in-depth analysis, it is necessary to obtain the solution to inverter output voltage $U_{\mathrm{out}}$ over the time *t* for a step pulse at the input. Afterward, an inverse function must be calculated for a given $U_{\mathrm{out}}$, i.e, $t_{pd}=t\left(U_{\mathrm{out}}=0.9u_{\mathrm{out}}\left(t=\infty \right),P\right)$, which allows for finding out the $t_{pd}$ relationship for different design variables $P=\left\{C,\;R_{\mathrm{in}}\;k_{u}\left(R_{k},\;W_{M_{1}},\;L_{M_{1}}\right)\right\}$.

**Figure 8:**
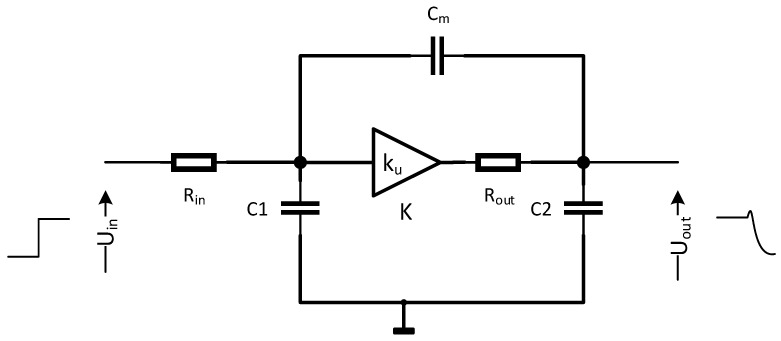
Nonlinear inverter macromodel.

One can see that the dimensions of the inverter design parameter space are too high for a reasonable and intuitive analysis. For this purpose, we conceived a simplified macromodel presented in Figure 8, which corresponds to the inverter in Figure 7b with the reduced parameter space. The voltage-controlled source *K* and $R_{\mathrm{out}}$ resistance are nonlinear, and both the macromodel circuit parameters $k_{u}\left(U_{\mathrm{in}}\right)$ and $R_{\mathrm{out}}\left(U_{\mathrm{out}}\right)$ depend on at least three different design variables ($R_{k},\;W_{M_{1}}$, and $\;L_{M_{1}}$) and three different operating regions of $M_{1}\ldots M_{3}$ (cut-off, triode, and pentode).

Moreover, the presence of $C_{1}$, $C_{2}$ (corresponding to the physical capacitances of the inverter) and $C_{M}$ requires describing the circuit with a set of nonlinear differential equations; hence, a simple symbolic solution explaining the influence of P over $t_{pd}$ is impossible. Therefore, we performed two methods of analysis in order to determine the optimum parameter set P for the maximum $t_{pd}$ sensitivity to the strain. In the first one, we used the Laplace transform of a small signal macromodel appropriate for the pentode region of the circuit presented in Figure 8.

In the second approach, we used the Cadence SPECTRE simulator, which allowed us to obtain nonlinear inverter transfer characteristics $U_{\mathrm{out}}\left(U_{\mathrm{in}}\right)$ (and $k_{u}\left(U_{\mathrm{in}}\right)=\frac{\Delta U_{\mathrm{out}}}{\Delta U_{\mathrm{in}}}{|}_{U_{\mathrm{in}}}$), and $R_{\mathrm{out}}\left(U_{\mathrm{out}}\right)$ output characteristics for various $R_{k},W_{M_{1}},L_{M_{1}}$. Afterward, we used spline approximations of $U_{\mathrm{out}}\left(U_{\mathrm{in}}\right)$ for *K* and $R_{\mathrm{out}}\left(U_{\mathrm{out}}\right)$ of the circuit in Figure 8 to build precise look-up tables (LUTs) for both stressed (ϵ=0.2%) and unstressed conditions and different values of $R_{k},\;W_{M_{1}}$, and $\;L_{M_{1}}$. Such an approach allowed us to reduce the dimensions of the design parameter space in which the analysis and optimization was performed. Therefore, we significantly improved the speed of analysis.

**Figure 9:**
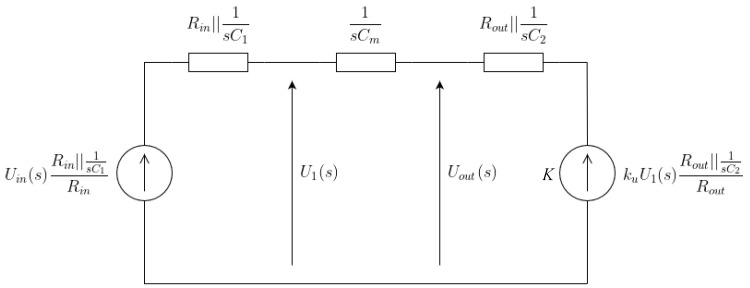
Linearized version of the inverter.

In order to perform the analysis of the circuit in Figure 8 with the Laplace transform, we linearized the circuit and transformed it to a less complex form. This led us to the circuit shown in Figure 9, in which $U_{\mathrm{in}}\left(s\right)$ corresponds to a Laplace transform of a Heaviside step function applied at the $U_{\mathrm{in}}$ input, $U_{1}\left(s\right)$ is an auxiliary variable controlling the linearized *K* source in Figure 8, and $U_{\mathrm{out}}\left(s\right)$ is the Laplace transform of the inverter’s step response. For our convenience, the circuit analysis of the input and output response with the Laplace transform was scaled down to −1 up to 1 V, whereas the results obtained with the inverse transform were scaled back to a quite typical voltage response, i.e, 0–3.3 V. The above circuit allowed us to derive a Laplace transform of the circuit transmittance (Equation (2)).

(2) $$\frac{U_{\mathrm{out}}\left(s\right)}{U_{\mathrm{in}}\left(s\right)}\;\;=\;\;\frac{sC_{m}R_{\mathrm{out}}+k_{u}}{sC_{m}\;\;\left(\;C_{2}sR_{\mathrm{in}}\;R_{\mathrm{out}}\;\;\;-\;\;\;R_{\mathrm{in}}k_{u}\;\;\;+\;\;\;R_{\mathrm{in}}\;\;\;+\;\;\;R_{\mathrm{out}}\;\right)\;\;+\;\;C_{1}sR_{\mathrm{in}}\;\left(\;sC_{m}R_{\mathrm{out}}\;\;\;+\;\;\;C_{2}sR_{\mathrm{out}}\;\;\;+\;\;\;1\;\right)\;\;+\;\;C_{2}sR_{\mathrm{out}}\;\;+\;\;1}$$

An inverse transform of Equation (2) is troublesome for a direct analysis; however, the complete equation describing $u_{\mathrm{out}}\left(t\right)$ step response is available in the Appendix A.1. With the use of $u_{\mathrm{out}}\left(t\right)$, we analyzed $t_{pd\;\mathrm{stressed}}$ and $t_{pd\;\mathrm{unstressed}}$ according to the relative parameter changes obtained in Section 2.1. In this way, we were able to perform data screening for various P and solve the optimization problem, i.e., $\max_{P}\frac{t_{pd\;\mathrm{stressed}}-t_{pd\;\mathrm{unstressed}}}{t_{pd\;\mathrm{unstressed}}}$, in order to choose the design parameter set, thereby, ensuring the highest inverter sensitivity to the mechanical stress.

The initial (unstressed) parameters taken as the starting points in the macromodel simulation (with the MATLAB Simulink environment) were $R_{\mathrm{out}}=145\;k\Omega$, $R_{\mathrm{in}}=355\;k\Omega$, $C_{m}=700\;\mathrm{fF}$, $C_{1}=33\;\mathrm{fF}$, and $C_{2}=62\;\mathrm{fF}$. The small signal gain was within the $k_{u}\in \left\{-8,\;-1.8\right\}$ limits.

The $R_{\mathrm{out}}$, $C_{1}$, and $C_{2}$ resulted from the physical parameters extracted with Cadence SPECTRE and Mentor Calibre PEX environments for an inverter with typical $M_{2}$ and $M_{3}$ sizes of L=800nm and W=5μm (see Figure 7b). The $R_{k}$ and $M_{1}$W/L ratios were chosen to obtain gains within the $k_{u}\in \left\{-8,\;-1.8\right\}$ range. The initial value of the $C_{m}$ capacitor was $C_{M}\approx 700\;\mathrm{fF}$, which corresponded to a 16×16μm square structure with a thin ZnO dielectric inside.

The initial $C_{M}\approx 700\;\mathrm{fF}$ ensured a previously assumed relatively low operating frequency of the ring oscillators (i.e., $f_{u}\in \left\{60\;\mathrm{kHz},\;150\;\mathrm{kHz}\right\}$), which allowed for easy measurements of the frequencies with an embedded counter/divider or a spectrum analyzer. In order to maximize the stress-induced influence on $M_{1}$ transconductance, according to the results presented in Section 2.1, we used the lowest possible W/L ratio (along with a matching $R_{k}$) that still ensured proper switching (noise margins) of the inverter.

**Figure 10:**
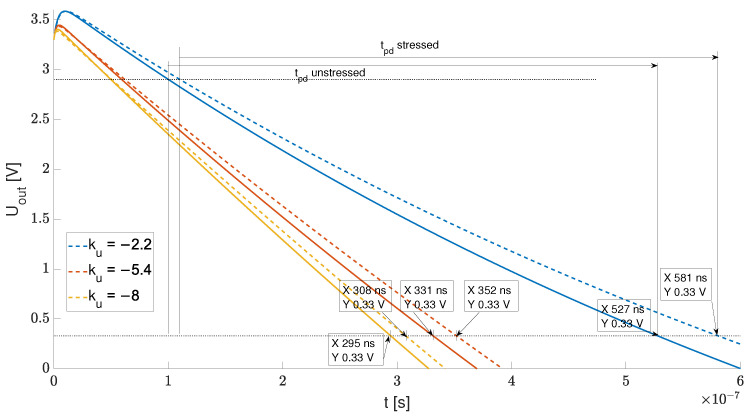
Example step responses and the influence of $k_{u}$ on $t_{pd}$ for stressed and unstressed devices.

The example $t_{pd}$ results obtained for the $k_{u}=\left\{-2.2,\;-5,4,\;-8\right\}$ set were calculated with both the linear system from Equation (2) and SPECTRE for nominal and stressed sets of parameters (ϵ≈0.2%). The results obtained with Equation (2) are shown in Figure 10. Both the nonlinear and linear analysis showed a significant increase of $|\frac{t_{pd\;\mathrm{stressed}}-t_{pd\;\mathrm{unstressed}}}{t_{pd\;\mathrm{unstressed}}}|>12\%$ for an inverter with the lowest $|k_{u}|$ in comparison to 5.2% and 7.4% for $k_{u}=-8$ and $k_{u}=-5.4$, respectively. One can see that, according to Figure 10, an additional low time constant reduces the slope of $u_{\mathrm{out}}\left(t\right)$ for low $|k_{u}|$, which additionally increases the stress impact on $t_{pd}$. Based on these initial findings, we performed a constrained optimization task, i.e., $\max_{P}\left|\frac{t_{pd\;\mathrm{stressed}}-t_{pd\;\mathrm{unstressed}}}{t_{pd\;\mathrm{unstressed}}}\right|$ for both the linear and nonlinear macromodels of the inverter.

For the purpose of the numerical optimization, we used a MATLAB *fmincon* function with the interior point algorithm [28]. The macromodel variables subjected to optimization were reduced to a vector of independent design parameters $P=\left\{k_{u},\;R_{\mathrm{in}},\;C_{m}\right\}$. We assumed that the $R_{\mathrm{out}}$ parameter is constant and solely depends on the dimensions of $M_{2}$ and $M_{3}$, which are set to the minimum allowed process dimensions; therefore, it was eliminated from P.

**Figure 11:**
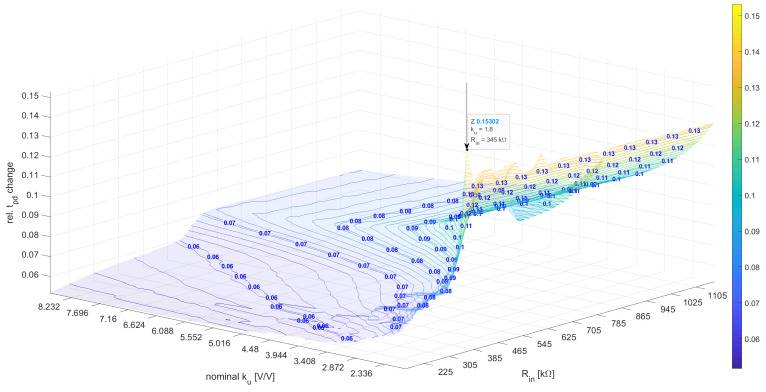
The relative $t_{pd}$ influence for various $k_{u}$ and $R_{\mathrm{in}}$ for a linear model.

The obtained optimum set for the linear model was $P_{\mathrm{lin}}=\left\{k_{u}=-1.8,\;R_{\mathrm{in}}=345\;k\Omega ,\;C_{m}=677.5\;\mathrm{fF}\right\}$, with the $k_{u}$ value reaching the optimization constraint —$|k_{u}|<1.8$, which led to an infeasible solution in which the inverter did not reach the logical zero at the output due to improper noise margins. Figure 11 shows the $|\frac{t_{pd\;\mathrm{stressed}}-t_{pd\;\mathrm{unstressed}}}{t_{pd\;\mathrm{unstressed}}}|$ dependence for the variables $k_{u}$ and $R_{\mathrm{in}}$ as well as the constant $C_{m}=690\;\mathrm{fF}$.

**Figure 12:**
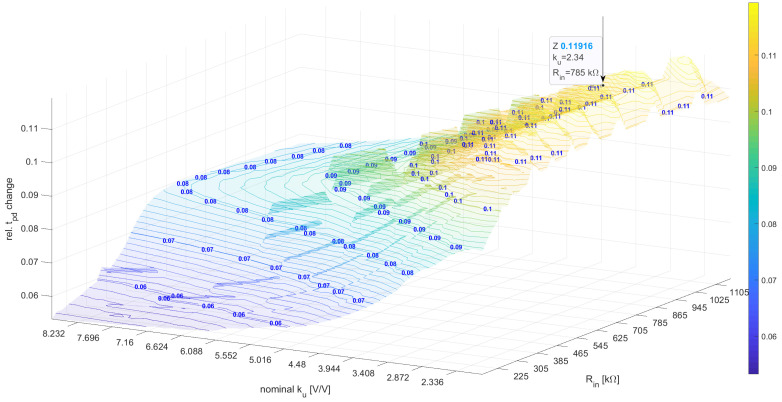
The relative $t_{pd}$ influence for various $k_{u}$ and $R_{\mathrm{in}}$ for a nonlinear model.

The optimization of $\max_{P}\left|\frac{t_{pd\;\mathrm{stressed}}-t_{pd\;\mathrm{unstressed}}}{t_{pd\;\mathrm{unstressed}}}\right|$ for the nonlinear macromodel brought slightly different results, i.e., $P_{\mathrm{nonlin}}=\left\{R_{\mathrm{in}}=785\;k\Omega ,\;k_{u}=-2.34,\;C_{m}=670\;\mathrm{fF}\right\}$. In this case, the algorithm did not reach any of the constraints; however, the optimum small signal gain was also low, i.e., $k_{u}=-2.34$. The $R_{\mathrm{in}}$ resistance was more than twice as high compared to the linear macromodel optimization results; however, the objective function had a very small derivative for $k_{u}=-2.34$ and the $0.4\;M\Omega <R_{\mathrm{in}}<\;1\;M\Omega$ range (see Figure 12).

The optimization results indicated a nontrivial solution in which the Miller capacitance, together with a low $|k_{u}|$, flatten the step response of the circuit, thereby, leading to a $t_{pd}$ increase. Moreover, a low $\frac{du_{\mathrm{out}}\left(t\right)}{dt}$ value near the $0.9u_{\mathrm{out}}\left(\infty \right)$ level enhances the inverter $t_{pd}$ sensitivity to stress-induced fluctuations of $R_{\mathrm{in}}$ and $k_{u}$.

According to these outcomes, we fabricated a frequency response sensor with the design parameters corresponding to the results obtained above according to the block diagram in Figure 6. The optimum set of macromodel parameters $P_{\mathrm{nonlin}}$ corresponded to a physical design parameter set, i.e., $P=\{W_{R_{\mathrm{in}}}=3$ μm, $L_{R_{\mathrm{in}}}=3.5$μm, $W_{R_{k}}=3$ μm, $L_{R_{k}}=10$ μm, $W_{M1}=L_{M1}=W_{M2}=W_{M3}=5$ μm, $L_{M2}=L_{M3}=0.8$ μm, and $W_{C_{M}}=L_{C_{M}}=16$ μm}. We used the maximum $L_{M1}$ that allowed us to obtain the given $k_{u}=-2.34$. In this way, according to results presented in Figure 4b, the highest stress-induced sensitivity is observed.

**Figure 13:**
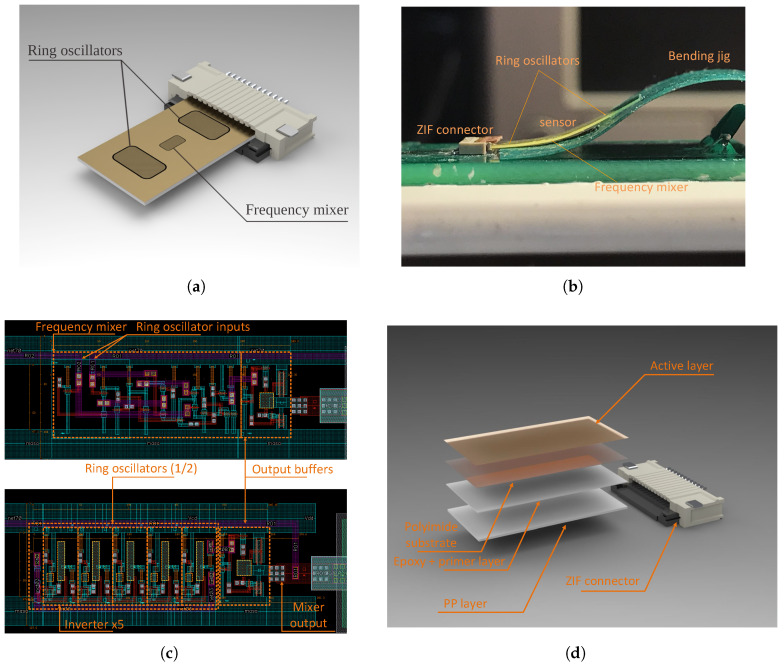
Mechanical assembly mode —SolidWorks bottom view (**a**), its physical implementation (side view) during tension measurements (**b**), internal sensor structure layouts (**c**), and (**d**) the complete sensor layer stack (bottom view).

The bottom and side views of the sensor with the corresponding layouts of the DFF and ring oscillators with the optimum physical parameter set (see Figure 13c) are shown in Figure 13a–c. The topology of both ring oscillators is identical; therefore, only a single layout is shown. The output signals of ring oscillators and DFF are buffered by two stages of inverters and attached to the ZIF connector, together with the power supply rails.

The whole sensor structure is 13mm long and 6.5mm wide and ends with a Molex 12-contact 500μm pitch ZIF connector soldered to a PCB [29], which acts as sensor housing. In order to increase the sensor sensitivity to the bending radius and to ensure a tight fit to the ZIF connector, we added a 180μm thick polypropylene (PP) layer, a 70μm thick adhesive primer, and an epoxy layer. The whole layer stack of the sensor is shown in Figure 13d. One can see that the active layer is placed at the bottom of the sensor, whereas its buffer layer is stuck to the polypropylene. The ring oscillators are intentionally placed in locations subjected to different strains to ensure a frequency difference resulting from the stress-induced $t_{pd}$ fluctuations.

## 3. Results

The physical experiment results that are described in this section required applying load forces (*F*) to the sensor, which resulted in the sensor bending with radii (*r*), and this varied along the sensor’s length. In this way, an ϵ(r) dependence was obtained in the physical proximity of both ($f_{s}$ and $f_{u}$) frequency oscillators. For this purpose, we simulated a deflection of the whole multi-layer sensor structure in the SolidWorks 2022 environment. We assumed that the sensor acts as a beam with a single end fixed support, in which a perpendicular point load (*F*) is applied to the other end. In this way, we were able to obtain the local strain distribution in the sensor.

**Figure 14:**
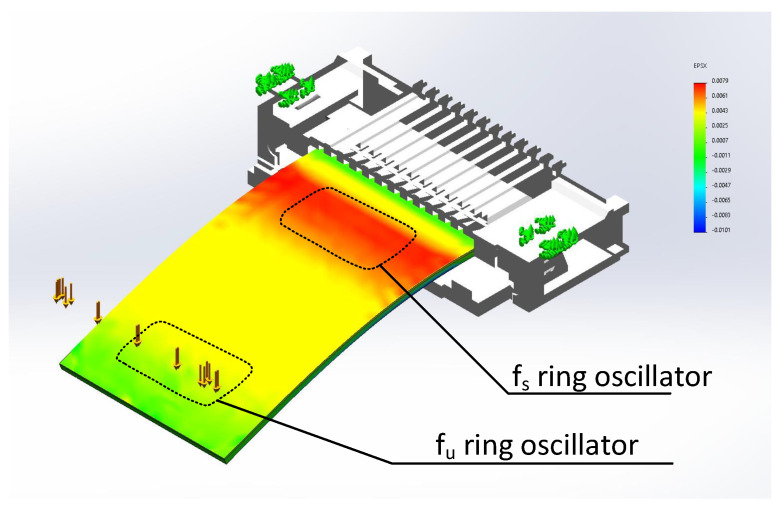
Mechanical stress simulation of the complete structure, bottom view. Renders in Figure 13a,d and Figure 14 based on Easy-On FFC/FPC Connector PN 505110-1297 STEP Model, courtesy of Molex LLC.

The distribution of ϵ in the sensor is responsible for differences of the inverters’ $t_{pd}$ values of the ring oscillators (see Figure 13a) as previously described in Section 2.1 and Section 2.2. In Figure 14, an example calculated bottom-side strain distribution is shown, in the case of a F=0.2N load applied at the sensor’s end. The example shows that the strain 2mm away from the fixing point (ZIF connector) at the active a-IGZO surface of the $f_{s}$ ring oscillator was ϵ≈0.008, whereas the strain near the other sensor end (where the second $f_{u}$ ring oscillator is located) was only ϵ≈0.0007 (over an order of magnitude lower).

The results clearly show that ϵ should primarily affect the material properties near the $f_{s}$ oscillator, whereas the vicinity of the $f_{u}$ oscillator should remain almost unchanged. The mechanical stress near the $f_{s}$ oscillator should result in a slight offset in the atomic distance and changes in the energy level splitting of the binding orbitals between the atoms of the semiconductor layer [22].

These changes affect the Fermi function, leading to a transistor $U_{t}$ shift, which, in turn, changes the transconductances ($g_{m}$) and, eventually, the inverters’ small signal gain (i.e., $k_{u}\left(\epsilon \right)\approx g_{M1m}\left(\epsilon \right)R_{k}\left(\epsilon \right)$; see the previous section). As discussed in Section 2.3, the small signal gain $k_{u}$ affects the performance (understood as the propagation delay $t_{pd}$) of inverters due to the presence of the Miller effect.

Concerning an obvious relationship describing the ring oscillator frequency of oscillation, i.e., $f_{s}=\frac{1}{2\cdot n\cdot t_{pd}}$ (where the length of the inverter ring in the proposed solution is n=5), one can see that $f_{s}$ is a stress-dependent variable. Moreover, the stress-induced variations of interatomic distances influence the electron-lattice interaction and lead to changes in the mobility μ. Therefore, the compressive strain decreases the electron mobility μ (as shown in Section 2.1), whereas the tensile strain causes the opposite behavior.

One can see that, according to Equation (1), the drain current $I_{D}$ is a mobility-dependent variable. Therefore, the final slew rate $\frac{dV(t)}{dt}=\frac{I_{D}\left(t\right)}{C_{\mathrm{in}}}$ of each inverter (and $t_{pd}$, which heavily depends on $\frac{dV}{dt}$; see Figure 5b), rises in cases of tensile stress and decreases for compressive stress. In this way, the $f_{s}$ depends on μ (and ϵ). In contrast, $f_{u}$ remains almost constant since it comes from a circuit that is an order of magnitude less affected by ϵ (and therefore almost constant μ) leading to a high non-zero $\Delta f=f_{s}-f_{u}$ as expected.

**Figure 15:**
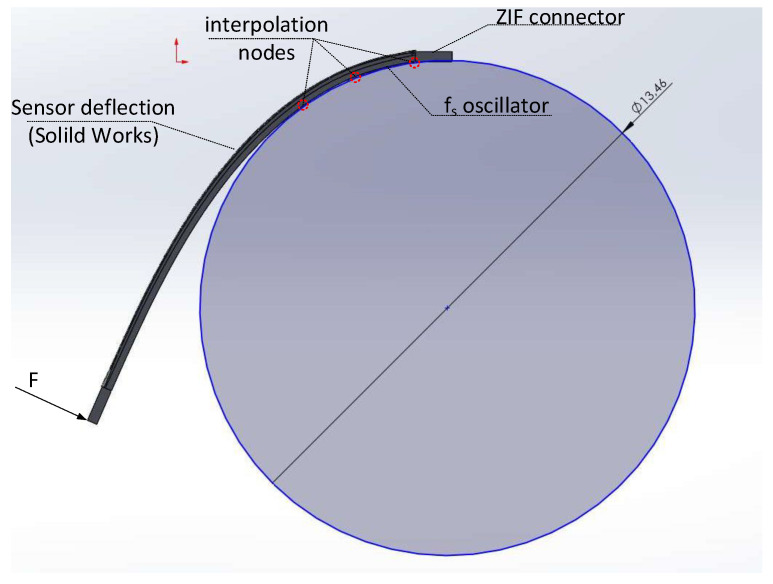
Local sensor deflection approximation near the $f_{s}$ oscillator obtained with finite element analysis.

As the strain near the $f_{u}$ oscillator can be regarded as negligible, and the strain near the $f_{s}$ oscillator correlates with the ϵ and local radius *r* of curvature near the connector, in the following section of the paper, we focus solely on the local curvature radius near the connector. The example SolidWorks-based simulation and local approximation (with a circle of a matching diameter) of a sensor deflection is depicted in Figure 15.

The use of an additional PP layer increased the strain ϵ obtained for the corresponding bending radii *r*. Therefore, the measurements of the Δf(ϵ) relationship obtained with a physical structure can be more accurate, i.e, higher bending radii were necessary to obtain ϵ corresponding to higher Δf. For the purpose of sensor characterization, we applied a bending force at the end of the sensor structures and measured the obtained radii *r* and Δf. The tensile force caused the bottom layers (i.e., the active layer) to expand; therefore, the decrease in $t_{pd}$ and the increase of Δf was observed.

**Figure 16:**
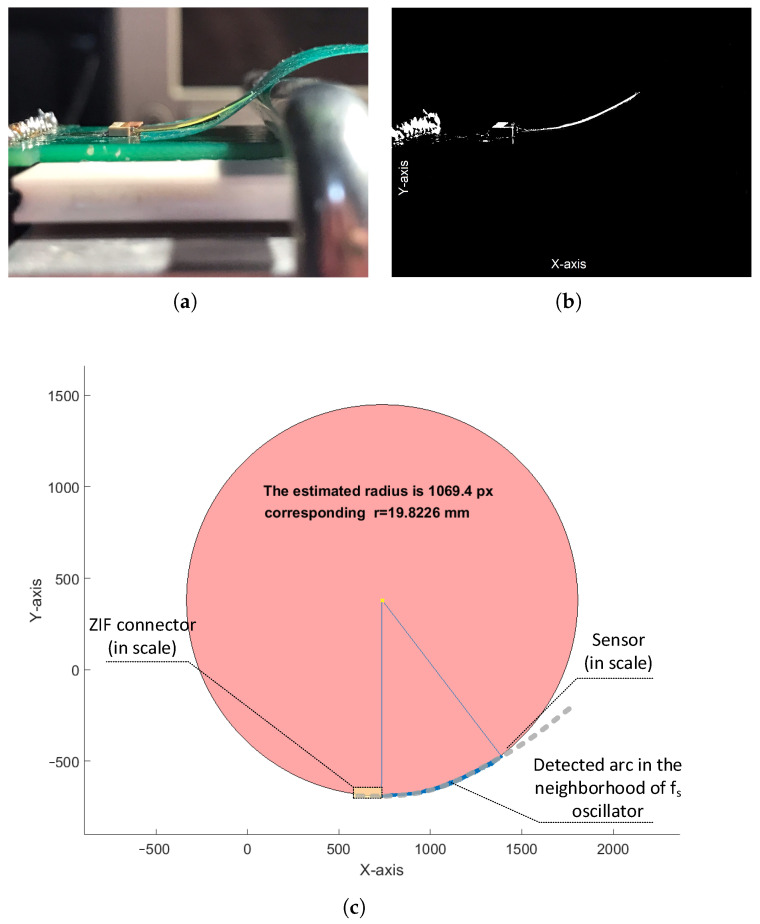
Arc detection algorithm (image processing) used in the radius estimation: test bench (**a**), picture after the edge detection (**b**), and (**c**) automated circle approximation (the sensor and ZIF connector are also marked in scale for greater readability).

The manufactured physical sensors structures were supplied from a 3.3V stabilized voltage supply by the ZIF connector, and the average power consumption was 0.4mW. The ring oscillators generated square waves (with $f_{s}$ and $f_{u}$ frequencies), which were applied to the DFF mixer. In this way, a periodic signal of Δf frequency(deviation) was measured at the output of the sensor’s mixer circuit. For this purpose, we used a Tektronix MDO3024 oscilloscope with the Fast Fourier Transform (FFT) analysis. In order to provide quick, accurate, and automated *r* measurements of the sensor arc near the ZIF connector, we used a picture analysis (edge detection) algorithm of the sensor test bench (Figure 16a shows the test bench).

Therefore, during each Δf measurement, we captured the shape of the bent sensor (arc) in proximity of the ZIF connector with a camera (Figure 16b shows the results of the edge-detection algorithm) instead of bending the sensor with cylinders of a reference radii, which was troublesome due to the ZIF connector housing. This approach increased both the accuracy and speed of the measurements. In order to calculate the arc radius, we used the least squares algorithm that approximated the detected arc of a bent sensor with a circle (Figure 16c shows the example results of automated circle fitting in the MATLAB environment). In the automated arc approximation algorithm, we used the arc shape placed closer to the ZIF connector, since the curvature of the bent sensor changes with the distance from the fixing point.

We measured *r* and Δf for 10 sensor structures that came from the same wafer. First, we tested the sensors for six different bending radii, i.e, r={ 7.5, 13, 16, 20, 23, and 29 mm }. Second, we estimated their maximum strains ϵ with the SolidWorks environment for corresponding *r*, 2mm from the ZIF connector where the $f_{s}$ ring oscillator is located. The maximum calculated strains for corresponding r were ϵ={ 0.0086, 0.0055, 0.004, 0.0033, 0.0029, 0.0021 }. To validate the results obtained with the image processing algorithm, we also propped up the circuit with a dynamometer gauge arm to measure the mechanical force exerted perpendicularly to the circuit surface.

**Figure 17:**
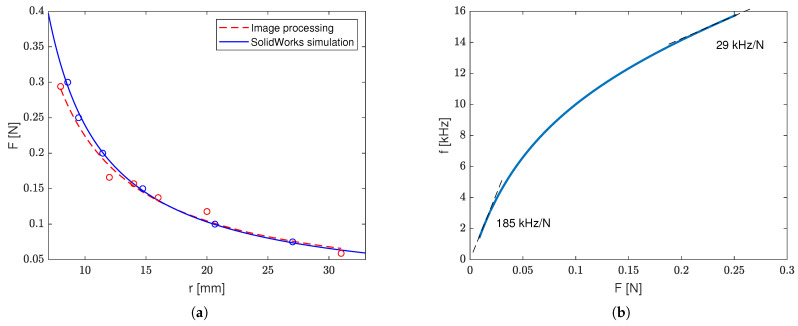
Sensor characterization results: (**a**) comparison of F(r) relationships obtained with SolidWorks and the physical measurements and (**b**) the output characteristic function of the sensor.

Figure 17a shows the relationship between the applied force and the resulting radii obtained via image processing compared with the same relationship taken from the SolidWorks simulation. After running a static simulation study for each force, the local radius of curvature near the $f_{s}$ oscillator was obtained by finding a circle passing through three points near the connector in each of the deflected Finite Element Analysis (FEA) meshes (see Figure 15).

**Figure 18:**
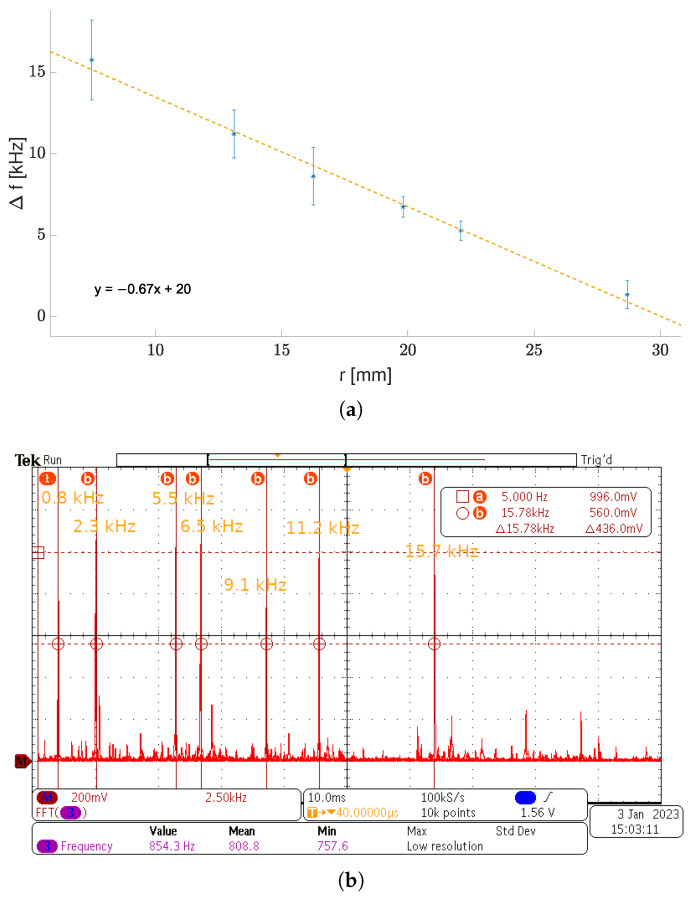
Frequency measurements of a physical sensor structure, output frequency vs. bending radius (**a**) and (**b**) the frequency–domain response of the sensor for variable radius.

One can see that the image-processing-based estimation gave results that are close to these obtained with SolidWorks. With the use of empirical relationships of Δf(r) (presented in Figure 18a) and r(F) (based on both numerical estimates and physical measurements), we were able to estimate the sensor output characteristic function, i.e., Δf(F), which is shown in Figure 17b. This function allowed us later to estimate such crucial sensor parameters as sensitivity, the limit of detection (LoD), and the limit of quantification (LoQ).

The Δf frequency measurement results are presented in Figure 18a, in which each point corresponds to an average value coming from multiple measurements of various sensor specimens. The Δf values were obtained with the spectrum analysis based on the FFT of the differential signal at the frequency mixer (DFF) output. Figure 18b shows an overlay of some of the measured oscillation peaks in the frequency domain (spectrum analysis of the signal from the DFF mixer) with their frequencies rising along with a decreasing *r*.

It is worth mentioning that the maximum and minimum $f_{s}$ frequencies (before mixing) were 91 and 76kHz, respectively, whereas $f_{u}$ remained almost unchanged ($f_{u}\approx 76\;\mathrm{kHz}$). The $f_{u}$ and $f_{s}$ difference (Δf), when no mechanical stress was applied, resulted from the local circuit mismatch. However, during measurements, this difference did not exceed 1.8kHz. The Δf uncertainty bounds in the picture show the minimum and maximum values obtained for each sensor specimen and its bending *r*. The linear approximation of Δf(r) dependence yielded an average $-0.67\;\frac{\mathrm{kHz}}{\mathrm{mm}}$ sensitivity coefficient; however, a Δf saturation was observable at low *r*.

**Figure 19:**
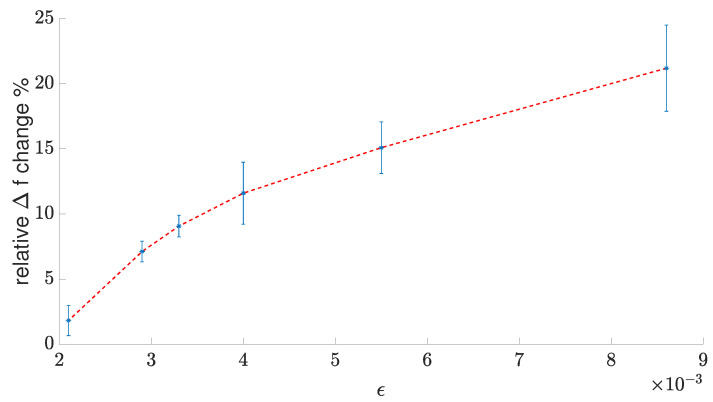
Relative frequency deviation vs. strain.

The saturation effect at low *r* (high ϵ) is clearly visible on the relative frequency deviation plot, i.e., $100\frac{\Delta f(\epsilon )}{f_{u}}\%$, which is shown in Figure 19.

One can see that the initial sensitivity of the relative frequency deviation at low ϵ<0.003 is almost three-times higher than for ϵ>0.005. The frequency deviation for relatively low ϵ is mainly caused by the $U_{T}$ and $K_{n}$ fluctuations near its nominal values for the unstressed material. The deviation saturation for higher ϵ likely results from the maximum available mobility μ of the material for a certain interface trap density, which, in turn, depends highly on the mechanical strain [13].

In the proposed structure, the strain resulting from the applied force *F* causes the opposite parameter change to the one described in Section 2.1. In the setup discussed in Section 2 we investigated the transistor parameter fluctuation resulting from the material compression, whereas, in this case, the tensile forced was applied to resistors and transistors forming the ring oscillators. The saturation of electron mobility for higher strains decreased the strain-induced impact of $I_{D}$, thereby, decreasing the sensor’s sensitivity for high ϵ. Therefore, the sensor characteristic curve, i.e., S=Δf(F), is a nonlinear function of force *F*, and thus the sensor manifests different sensitivities in different ranges of operation.

The higher strains, i.e., ϵ>0.01 sometimes caused permanent degradation effects on a few sensors. Such effects resulted in a sudden and permanent frequency drop or output signal deterioration, e.g., aperiodic responses of the ring oscillators. This behavior may result from contact resistance degradation by the local delamination or the increase in the access resistance resulting from the defect formation. An additional explanation for this phenomenon may result from the formation of cracks in the material [30]. Strain applied in parallel to the source-drain current path (i.e., the channel of the NMOS transistors) may cause severe degradation when ϵ>0.0085.

The degradation occurs as cracks in the transistor channel cause a bottleneck effect on the flow of electrical current [30]. Therefore, for high strains, damage formation limits the $I_{D}$ current leading to saturation of the stress-induced Δf (and $f_{s}$) increase. Moreover, hysteresis effects may appear for ϵ>0.0085, as not all the cracks will disappear after re-flattening the sensor (i.e., applying ϵ=0). For this reason, we assumed that the maximum acceptable and repeatable ϵ that does not affect the material degradation is less than 0.0086, which, in the case of our sensor, corresponds to F<0.3N and a 7.5 mm bending radius. The occurrence of this effect defined an obvious bound for the measurement range of our sensor.

Based on the measurements of the bending radii (*r*), force (*F*), and SolidWorks ϵ(F) strain estimates (presented in Figure 17a, Figure 18a, and Figure 19), we were able to obtain the output characteristic curve S=Δf(F) of the sensor (presented in Figure 17b) and performance measures, such as the accuracy (Accu), precision (Prec), sensitivity ($\frac{dS}{dF}$), resolution (Res), limit of detection (LoD), and limit of quantification (LoQ). Accuracy measurements were based on the inverse function $S^{-1}:\Delta f\to F$ of the sensor characteristic curve, the physical bending force measurements, and corresponding $\hat{\Delta f}$ frequencies. In this way, the differences between the physically applied force *F* and its estimates $\hat{F}=S^{-1}\left(\hat{\Delta f}\right)$ were found. The estimated accuracy was Accu=8%, whereas the precision (based on the scatter of measured $\hat{F}$ values) was Prec=0.023N.

The proposed sensor, despite its digital design, utilizes fully analog phenomena. The sensor’s output quantity (frequency deviation Δf) is a result of a relative position of rising slopes of square wave signals from the $f_{s}$ and $f_{u}$ ring oscillators. Therefore, Δf is a continuous variable, and the sensor can be considered an analog sensor. For this reason, in order to estimate the sensor resolution, it was necessary to estimate the sensor noise, resulting from the Δf momentary fluctuations and measurement range.

Considering the measured $\hat{\Delta f}$ standard deviation, i.e., $\mathrm{std}\hat{\Delta f}=0.2\;\mathrm{kHz}$, and the sensor measurement range, i.e., 0.3N, the resultant resolution was Res=0.004N. The sensor characteristic transfer curve is nonlinear; therefore, its sensitivity varies with the slope. The minimum sensitivity was $\frac{dS}{dF}=29\;\frac{\mathrm{kHz}}{N}$, whereas the maximum sensitivity was $\frac{dS}{dF}=185\;\frac{\mathrm{kHz}}{N}$. Both the limit of detection and quantification were estimated by using the calibration curve *S* method. In this method, based on the standard deviation of the output quantity, i.e., $\mathrm{std}\hat{\Delta f}$, the *y*-axis intercept points at the calibration curve were found for 3.3σ (in the case of LoD) and 10σ (in the case of LoQ) as well as their corresponding measured quantity values (i.e., *F*). Based on this method, the estimated LoD and LoQ were 0.0099N and 0.030N, respectively, allowing us to estimate the minimum of the proposed sensor’s range.

**Figure 20:**
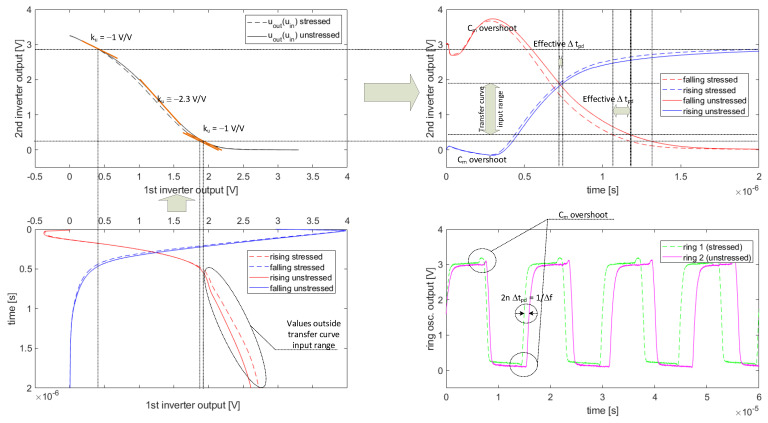
$\Delta t_{pd}$ masking mechanism.

The other important observation during the measurements was that, despite the relatively high sensitivity of the device to mechanical stress, the initial relative frequency deviation for low strains, i.e., ϵ≈0.002, was below expectation (≈2%) when compared to values of the optimization criterion denoted in Section 2.3 as $|\frac{t_{pd\;\mathrm{stressed}}-t_{pd\;\mathrm{unstressed}}}{t_{pd\;\mathrm{unstressed}}}|$. Due to the voltage shift of the inverter transfer curve towards low input voltages, the inverters were immune to excessively long rising slopes and more vulnerable to falling ones. The diagram presented in Figure 20 explains the mechanism of so-called $\Delta t_{pd}$ flattening for edges of a certain type in subsequent inverters of a ring.

The edges of the first inverter output waveforms (intentionally rotated clockwise at the bottom-left part of Figure 20) are passed to the transfer curve of the second inverter (see Figure 6). One can see that the highest differences between the stressed and unstressed cases are visible for the rising slope. These differences are mostly visible for low $U_{GS}$ voltages of the M2 voltage follower (see Figure 7b), which manifests low transconductance in this region of operation. The higher sensitivity to ϵ for low transconductances was previously discussed in Section 2.1.

Moreover, low transconductance (resulting in high $r_{ds}$ of M2) reduces the speed $\frac{dV}{dt}$ of $C_{M}$ charging, which additionally decreases the output slew rate (see both rising slopes in the bottom-left waveform of Figure 20). Unfortunately, the majority of this low $\frac{dV}{dt}$ region, where a high sensitivity to stress is observed, is masked by a low $|k_{u}|\to 0$ (see the top-left transfer curve of the diagram in Figure 20) because this particular region is mainly situated outside the active input range of the transfer curve.

Nevertheless, despite the described $\Delta t_{pd}$ flattening effect, one can see that the resultant response of the second inverter manifests high $\Delta t_{pd}$. Unfortunately, in the case of the falling edge at the first inverter output, a high $M_{3}$ transconductance (caused by its high $U_{GS}$) masks the stress impact on the falling edge.

This falling edge passed to the transfer curve of the consecutive inverter turns into the rising edge, which manifests a slight $\Delta t_{pd}$ sensitivity—mainly due to the transfer curve shift caused by the stress-induced $U_{t}$ shift (see the top-left side of the diagram in Figure 20). One can see that, despite the high influence of stress on falling/rising slopes near the asymptotic values (0 and 3.3V), the effective $\Delta t_{pd}$ (within nonzero $k_{u}$ bounds) is smaller.

We also experimentally verified the influence of small signal gain and the Miller effect on the sensitivity of the sensor. For this purpose, we fabricated six different sensor versions with/without the Miller capacitor $C_{M}$ and three combinations of small signal gains $k_{u}=\left\{-8,\;-5,\;-2.3\right\}$. We measured the relative frequency deviation $\frac{\Delta f(\epsilon )}{f_{u}}$ for ϵ=0.0029 (r=23mm), which is one of the measurement points depicted in Figure 18a and Figure 19. The relative frequency deviations for various design parameter combinations are listed in Table 2.

One can see that physical measurements listed in Table 2 confirm the results obtained with macromodels, and the Miller effect improves the circuit performance, whereas the circuit with $k_{u}=-2.3$ yields the highest sensitivity.

## 4. Discussion and Conclusions

In the paper, we discussed the design and optimization of the a-IGZO stress sensor. For optimization purposes, we derived linear and nonlinear macromodels of inverters comprising the crucial part of the sensor. Both macromodels yielded similar results, showing that circuits with low gain and additional Miller effect bring the best sensitivity to electrical changes induced by mechanical stress. Over the whole measured range, i.e., ϵ=0 for an unstressed sensor up to ϵ=0.86% strain (corresponding to a 7.5 mm bending radius), the physical sensor structure exhibited a 20% frequency deviation (i.e., 16kHz) in the output signal.

For the sensor characterization, we used an image processing algorithm that automatically calculated the arc radius of the device under test. The sensor is of a differential structure, which makes it immune to power supply and temperature variations. Its digital output facilitates further integration with digital blocks contained within the same system. Moreover, it does not require sophisticated analog subcircuits for signal conditioning, whereas the measured quantity (strain or force) corresponds to a digital signal frequency.

One of the disadvantages of the proposed design is the initial differential offset frequency for a sensor with no mechanical stress applied. This nonzero offset results from the local mismatches and, during measurements, did not exceed 1.8kHz, thus, corresponding to a 2.3% error of the average output frequency for a non-stressed device. Such an inconvenience can be easily removed by an offset reset applied by a microcontroller device during circuit warm-up. The other drawback may result from the flexible nature itself, i.e., elasticity in smart packaging, where the whole product and its packaging are susceptible to deformation. In such a case, the packaging would likely have to be designed from the ground up with the sensor in mind.

However, flexible packaging constitutes a tiny part of the smart packaging market. There might be another niche for goods that are sensitive to deformation while at the same time being infeasible to store in rigid containers. The main advantages of the proposed sensor are its high sensitivity ($185\;\frac{\mathrm{kHz}}{N}$) and resolution (0.004N) resulting from the nonlinear strain vs. material mobility relationship, with a low power consumption of 0.4mW maintained during a measurement.

Some hysteresis is to be expected for ϵ>0.85% due to the formation of cracks in the material; however, it proved difficult to quantify using our measurement methods for ϵ<0.85% within the sensor’s measurement range. Moreover, the stand-alone sensor (without the ZIF connector) can be considered as a 5 × 0.2 mm stripe, which, in a volume production, can cost less than half of a cent in USD. However, only about 6% of this sensor area would be used by the sensor layout. Considering the sensor as a part of a larger device (μC, RFID tag, etc.), the sensor footprint itself would cost around 3/100th of a cent. 

## Figures and Tables

**Table 1:** $K_{n}$ and threshold voltages of transistors in the pristine state (unstressed) and subjected to mechanical stress (compressive) strain ϵ=0.2%).

$\frac{W}{L}$ Ratio	Unstressed		Stressed	
	$U_{T}\;\left[V\right]$	$K_{n}\;\left[\frac{\mathrm{mA}}{V^{2}}\right]$	$U_{T}\;\left[V\right]$	$K_{n}\;\left[\frac{\mathrm{mA}}{V^{2}}\right]$
3/0.8	1.468	0.769	1.473	0.765
30/0.8	1.445	7.328	1.455	7.324
300/0.8	1.415	40.62	1.429	40.42
3/8	0.137	0.0264	0.238	0.0254
3/80	0.538	0.00825	0.595	0.00691

**Table 2:** Comparison of relative frequency deviations for optimum and non-optimum sets of inverter parameters both with and without the Miller capacitor for ϵ=0.0029 (r=23mm).

↓Deviation\Gain→	$k_{u}=-8$	$k_{u}=-5$	$k_{u}=-2.3$
with $C_{M}$			
$\frac{\Delta f}{f_{u}}$	0.0460	0.0473	0.0778
without $C_{M}$			
$\frac{\Delta f}{f_{u}}$	0.0538	0.0381	0.0098

## Data Availability

The data that support the findings of this study are available from the corresponding author, upon a reasonable request. Some data, concerning the technical details of the semiconductor technology used, may be subject to intellectual property restrictions..

## Appendix A

### Appendix A.1. Step Response of the Inverter

$$\begin{array}{c} u_{\mathrm{out}}\left(t\right)=k_{u}-\frac{e^{-\frac{tA_{3}}{A_{1}}}\left(\cosh\left(A_{4}\right)-\frac{R_{\mathrm{in}}R_{\mathrm{out}}\sinh\left(A_{4}\right)\left(\frac{A_{3}}{A_{1}}-\frac{C_{1}R_{\mathrm{in}}k_{u}-C_{m}R_{\mathrm{out}}+C_{2}R_{\mathrm{out}}k_{u}+C_{m}R_{\mathrm{in}}k_{u}+C_{m}R_{\mathrm{out}}k_{u}-C_{m}R_{\mathrm{in}}k_{u}{}^{2}}{A_{2}}\right)A_{5}}{B}\right)A_{2}}{C_{1}C_{2}R_{\mathrm{in}}R_{\mathrm{out}}+C_{1}C_{m}R_{\mathrm{in}}R_{\mathrm{out}}+C_{2}C_{m}R_{\mathrm{in}}R_{\mathrm{out}}}\\ where\\ A_{1}=2C_{1}C_{1}R_{\mathrm{in}}R_{\mathrm{out}}+2C_{1}C_{m}R_{\mathrm{in}}R_{\mathrm{out}}+2C_{2}C_{m}R_{\mathrm{in}}R_{\mathrm{out}}\\ A_{2}=C_{1}C_{2}R_{\mathrm{in}}R_{\mathrm{out}}k_{u}+C_{1}C_{m}R_{\mathrm{in}}R_{\mathrm{out}}k_{u}+C_{2}C_{m}R_{\mathrm{in}}R_{\mathrm{out}}k_{u}\\ A_{3}=C_{1}R_{\mathrm{in}}+C_{2}R_{\mathrm{out}}+C_{m}R_{\mathrm{in}}+C_{m}R_{\mathrm{out}}-C_{m}R_{\mathrm{in}}k_{u}\\ A_{4}=\frac{Bt}{R_{\mathrm{in}}R_{\mathrm{out}}A_{5}}\\ A_{5}=C_{1}C_{2}+C_{1}C_{m}+C_{2}C_{m} \end{array}$$

$$\begin{array}{c} B^{2}=\frac{{C_{1}}^{2}\;{R_{\mathrm{in}}}^{2}}{4}-\frac{C_{1}\;C_{2}\;R_{\mathrm{in}}\;R_{\mathrm{out}}}{2}-\frac{C_{1}\;C_{m}\;{R_{\mathrm{in}}}^{2}\;k_{u}}{2}+\frac{C_{1}\;C_{m}\;{R_{\mathrm{in}}}^{2}}{2}-\frac{C_{1}\;C_{m}\;R_{\mathrm{in}}\;R_{\mathrm{out}}}{2}+\frac{{C_{2}}^{2}\;{R_{\mathrm{out}}}^{2}}{4}\\ -\frac{C_{2}\;C_{m}\;R_{\mathrm{in}}\;R_{\mathrm{out}}\;k_{u}}{2}-\frac{C_{2}\;C_{m}\;R_{\mathrm{in}}\;R_{\mathrm{out}}}{2}+\frac{C_{2}\;C_{m}\;{R_{\mathrm{out}}}^{2}}{2}+\frac{{C_{m}}^{2}\;{R_{\mathrm{in}}}^{2}\;{k_{u}}^{2}}{4}-\frac{{C_{m}}^{2}\;{R_{\mathrm{in}}}^{2}\;k_{u}}{2}\\ +\frac{{C_{m}}^{2}\;{R_{\mathrm{in}}}^{2}}{4}-\frac{{C_{m}}^{2}\;R_{\mathrm{in}}\;R_{\mathrm{out}}\;k_{u}}{2}+\frac{{C_{m}}^{2}\;R_{\mathrm{in}}\;R_{\mathrm{out}}}{2}+\frac{{C_{m}}^{2}\;{R_{\mathrm{out}}}^{2}}{4} \end{array}$$

## References

[1] Barlian A.A., Park W.T., Mallon J.R., Rastegar A.J., Pruitt B.L. (2009). Review: Semiconductor Piezoresistance for Microsystems. Proc. IEEE.

[2] Lau W., Yang P., Siah S., Chan L. (2012). The role of a tensile stress bias for a sensitive silicon mechanical stress sensor based on a change in gate-induced-drain leakage current. Microelectron. Reliab..

[3] Song J., Huang X., Han C., Yu Y., Su Y., Lai P. (2021). Recent Developments of Flexible InGaZnO Thin-Film Transistors. Phys. Status Solidi A.

[4] Seo Y., Jeong H.S., Jeong H.Y., Park S., Jang J.T., Choi S., Kim D.M., Choi S.J., Jin X., Kwon H.I. (2019). Effect of Simultaneous Mechanical and Electrical Stress on the Electrical Performance of Flexible In-Ga-Zn-O Thin-Film Transistors. Materials.

[5] Billah M.M., Hasan M.M., Jang J. (2018). Millisecond Stretched Exponential Recovery of Threshold Voltage for Mechanically Stressed Flexible a-IGZO Thin-Film Transistors. IEEE Electron Device Lett..

[6] Billah M.M., Hasan M.M., Jang J. (2017). Effect of Tensile and Compressive Bending Stress on Electrical Performance of Flexible a-IGZO TFTs. IEEE Electron Device Lett..

[7] Park I.J., Jeong C.Y., Cho I.T., Lee J.H., Cho E.S., Kwon S.J., Kim B., Cheong W.S., Song S.H., Kwon H.I. (2012). Fabrication of amorphous InGaZnO thin-film transistor-driven flexible thermal and pressure sensors. Semicond. Sci. Technol..

[8] Xin C., Chen L., Li T., Zhang Z., Zhao T., Li X., Zhang J. (2018). Highly Sensitive Flexible Pressure Sensor by the Integration of Microstructured PDMS Film With a-IGZO TFTs. IEEE Electron Device Lett..

[9] Geng D., Han S., Seo H., Mativenga M., Jang J. (2017). Piezoelectric Pressure Sensing Device Using Top-Gate Effect of Dual-Gate a-IGZO TFT. IEEE Sens. J..

[10] Qiu A., Li P., Yang Z., Yao Y., Lee I., Ma J. (2019). A Path Beyond Metal and Silicon:Polymer/Nanomaterial Composites for Stretchable Strain Sensors. Adv. Funct. Mater..

[11] Zhang J.X.J., Hoshino K. (2019). Mechanical transducers: Cantilevers, acoustic wave sensors, and thermal sensors. Mol. Sensors Nanodevices.

[12] Schörner R., Poppinger M., Eibl J. (1990). Silicon pressure sensor with frequency output. Sens. Actuators A Phys..

[13] Jeong H.J., Han K.L., Ok K.C., Lee H.M., Oh S., Park J.S. (2017). Effect of mechanical stress on the stability of flexible InGaZnO thin-film transistors. J. Inf. Disp..

[14] Hsieh H.H., Kamiya T., Nomura K., Hosono H., Wu C.C. (2008). P-29: Modeling of Amorphous Oxide Semiconductor Thin Film Transistors and Subgap Density of States. SID Symp. Dig. Tech. Pap..

[15] Biggs J., Myers J., Kufel J., Ozer E., Craske S., Sou A., Ramsdale C., Williamson K., Price R., White S. (2021). A natively flexible 32-bit Arm microprocessor. Nature.

[16] Myny K. (2018). The development of flexible integrated circuits based on thin-film transistors. Nat. Electron..

[17] Dembo H., Kurokawa Y., Ikeda T., Iwata S., Ohshima K., Ishii J., Tsurume T., Sugiyama E., Yamada D., Isobe A. (2005). RFCPUs on glass and plastic substrates fabricated by TFT transfer technology. Proceedings of the IEEE InternationalElectron Devices Meeting, 2005—IEDM Technical Digest.

[18] Takayama T., Ohno Y., Goto Y., Machida A., Fujita M., Maruyama J., Kato K., Koyama J., Yamazaki S. (2005). A CPU on a plastic film substrate. Digest of Technical Papers, Proceedings of the 2004 Symposium on VLSI Technology, Honolulu, HI, USA, 15–17 June 2004.

[19] Nomura K., Ohta H., Takagi A., Kamiya T., Hirano M., Hosono H. (2004). Room-temperature fabrication of transparent flexible thin-film transistors using amorphous oxide semiconductors. Nature.

[20] Zhang L., Yu H., Xiao W., Liu C., Chen J., Guo M., Gao H., Liu B., Wu W. (2022). Strategies for Applications of Oxide-Based Thin Film Transistors. Electronics.

[21] Nathan A., Kumar A., Sakariya K., Servati P., Sambandan S., Striakhilev D. (2004). Amorphous silicon thin film transistor circuit integration for organic LED displays on glass and plastic. IEEE J. Solid-State Circuits.

[22] Münzenrieder N.S., Cherenack K.H., Tröster G. (2011). Testing of flexible InGaZnO-based thin-film transistors under mechanical strain. Eur. Phys. J. Appl. Phys..

[23] Jin J.W., Nathan A., Barquinha P., Pereira L., Fortunato E., Martins R., Cobb B. (2016). Interpreting anomalies observed in oxide semiconductor TFTs under negative and positive bias stress. AIP Adv..

[24] Chen Y., Geng D., Lin T., Mativenga M., Jang J. (2016). Full-Swing Clock Generating Circuits on Plastic Using a-IGZO Dual-Gate TFTs With Pseudo-CMOS and Bootstrapping. IEEE Electron Device Lett..

[25] Tiwari B., Martins J., Kalla S., Kaushik S., Santa A., Bahubalindruni P.G., Tavares V.G., Barquinha P. (2018). A High Speed Programmable Ring Oscillator Using InGaZnO Thin-Film Transistors. Proceedings of the 2018 International Flexible Electronics Technology Conference (IFETC).

[26] Hwang T.H., Yang I.S., Kwon O.K., Ryu M.K., Byun C.W., Hwang C.S., Park S.H.K. (2011). Inverters Using Only N-Type Indium Gallium Zinc Oxide Thin Film Transistors for Flat Panel Display Applications. Jpn. J. Appl. Phys..

[27] Zhou J., Kinniment D., Russell G., Yakovlev A. (2006). A robust synchronizer. Proceedings of the IEEE Computer Society Annual Symposium on Emerging VLSI Technologies and Architectures (ISVLSI’06).

[28] Villaverde A.F., Fröhlich F., Weindl D., Hasenauer J., Banga J.R. (2019). Benchmarking optimization methods for parameter estimation in large kinetic models. Bioinformatics.

[29] Jasanuma (2019). Easy-On FFC/FPC Connector, 0.50 mm Pitch, FD19 Series, Right-Angle, Bottom Contact, 1.90 mm Height, 12 Circuits, 150 °C Operating Temperature, Gold over Nickel Plating.

[30] Lee S.M., Shin D., Yun I. (2017). Degradation Mechanisms of Amorphous InGaZnO Thin-Film Transistors Used in Foldable Displays by Dynamic Mechanical Stress. IEEE Trans. Electron Devices.

